# Crystal Structure of Botulinum Neurotoxin Type A in Complex with the Cell Surface Co-Receptor GT1b—Insight into the Toxin–Neuron Interaction

**DOI:** 10.1371/journal.ppat.1000129

**Published:** 2008-08-15

**Authors:** Pål Stenmark, Jérôme Dupuy, Akihiro Imamura, Makoto Kiso, Raymond C. Stevens

**Affiliations:** 1 Department of Molecular Biology, The Scripps Research Institute, La Jolla, California, United States of America; 2 Institute for Integrated Cell-Material Sciences (iCeMS), Kyoto University, Yoshida, Sakyo-ku, Kyoto, Japan; The Rockefeller University, United States of America

## Abstract

Botulinum neurotoxins have a very high affinity and specificity for their target cells requiring two different co-receptors located on the neuronal cell surface. Different toxin serotypes have different protein receptors; yet, most share a common ganglioside co-receptor, GT1b. We determined the crystal structure of the botulinum neurotoxin serotype A binding domain (residues 873–1297) alone and in complex with a GT1b analog at 1.7 Å and 1.6 Å, respectively. The ganglioside GT1b forms several key hydrogen bonds to conserved residues and binds in a shallow groove lined by Tryptophan 1266. GT1b binding does not induce any large structural changes in the toxin; therefore, it is unlikely that allosteric effects play a major role in the dual receptor recognition. Together with the previously published structures of botulinum neurotoxin serotype B in complex with its protein co-receptor, we can now generate a detailed model of botulinum neurotoxin's interaction with the neuronal cell surface. The two branches of the GT1b polysaccharide, together with the protein receptor site, impose strict geometric constraints on the mode of interaction with the membrane surface and strongly support a model where one end of the 100 Å long translocation domain helix bundle swing into contact with the membrane, initiating the membrane anchoring event.

## Author Summary

Botulinum neurotoxins are the most toxic substances known and are classified as a category A bioterrorism agent. Ongoing work on the development of countermeasures for the neurotoxin has been limited by an incomplete understanding of the means by which the toxin enters the cell. Our study provides a detailed look at how the toxin binds its ganglioside co-receptor on the cell surface. Together with earlier work this generates a detailed description of how the toxin binds its two co-receptors to position it for entrance into the neuronal cell. This structural data provides critical new insight about the action of the botulinum neurotoxins that can be applied toward the development of agents to block toxin uptake in the digestive system and/or inhibit the binding of the toxin at the neuromuscular junction.

## Introduction

Botulism is a neuroparalytic disorder which is caused by botulinum neurotoxin (BoNT). It has a lethal intravenous dose of 1–5 ng/kg [1],[2], and acts by blocking the release of acetylcholine at the neuromuscular junctions, paralyzing the affected muscles. Despite its high toxicity, numerous widely used medical applications of the toxin have emerged in recent years [3],[4]. BoNT is a protease which is produced by *Clostridium botulinum* as a 150 kDa protein which must be proteolytically cleaved to become active. Once the two chains are formed, the light chain (∼50 kDa) and the heavy chain (∼100 kDa), continue to be associated through extensive interactions, including a cysteine bond and the translocation domain belt [5]. Seven serotypes of BoNT (A–G) have been identified and isolated. Each serotype has a different specificity or host organism, with serotype A, B and E (BoNT/A, -B and -E) as the most common source of infection in humans. Additionally, the different toxin serotypes are believed to utilize different protein receptors to enter the target cell.

**Figure 1:**
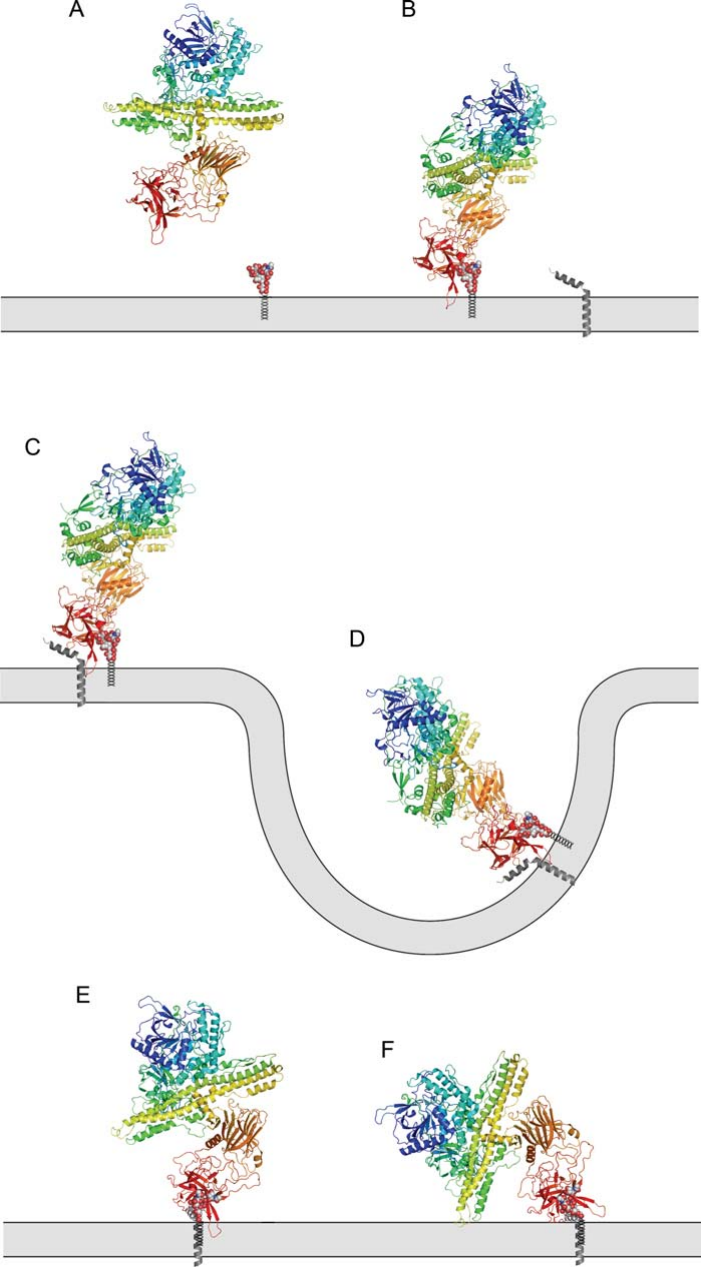
A model of the process of botulinum neurotoxin binding to the neuron surface. GT1b has been modeled into the BoNT/B Syt-II complex (2NP0) based on the BoNT/A binding domain-GT1b complex. Toxin displayed as rainbow colored ribbon, GT1b as CPK spheres and Syt-II as a gray ribbon. A: free toxin above the cell surface displaying GT1b. B: Toxin bound to GT1b on the cell surface. C: Toxin bound to GT1b and Syt-II on the neuron surface. D: Toxin entering the cell through endocytosis. E: Side view of the toxin along the axis of possible rotation. F: The N-terminal domain of the translocation domain (loops 590 and 750 in BoNT/B and loops 600 and 760 in BoNT/A) of the 100 Å long helixes from the translocation domain swinging into contact with the membrane inside the acidified endosome; it is also possible that the other end of the translocation domain make the initial contact with the membrane.

The holotoxin structures of BoNT/A and B have been solved [5],[6], and both structures contain three well-defined functional domains. In each, the C-terminal part of the heavy chain, the binding domain, interacts with specific gangliosides and protein receptors located on the presynaptic nerve terminals leading to endocytosis of the neurotoxin (Figure 1). SV2 has been proposed to be a protein receptor for BoNT/A [7], but it is possible that other receptors are involved, while Synaptotagmin I and II (Syt-I and -II) have been identified as protein receptors for BoNT/B and BoNT/G [8],[9],[10],[11]. Thus far, the only toxin-protein receptor complex that has been determined is the structure of BoNT/B in complex with the recognition domain of the Syt-II receptor [8],[9].

**Figure 2:**
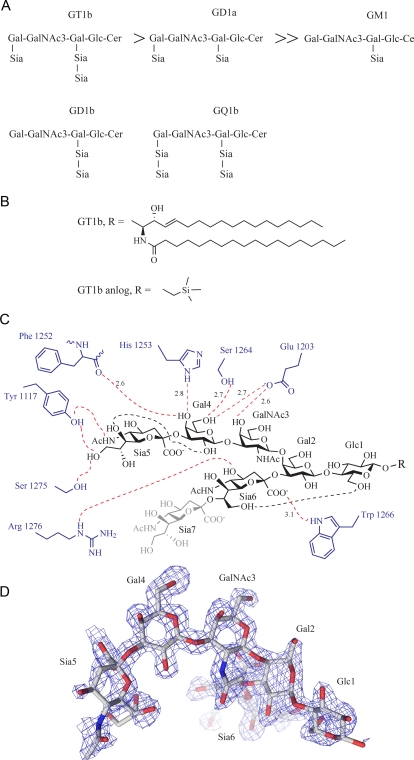
Gangliosides, GT1b and its interactions with BoNT/A. A: GT1b, GD1a and GM1 shown in decreasing order of affinity towards BoNT/A; also GD1b and GQ1b are shown. B: In GT1b a ceramide is present at the R position. In the GT1b analog used here; a CH_2_CH_2_Si(Me)_3_ group replaces the ceramide. C: Schematic picture of GT1b and its hydrogen bonds to BoNT/A. The hydrogen bonds between the protein (blue) and GT1b (black) are shown as dotted red lines and the GT1b internal hydrogen bonds as dotted black lines. Distances of key hydrogen bonds are displayed in Å. Sia7 that is disorderd in the complex is shaded gray. Numbered monosaccharide names are shown; Glc = glucose; Gal = Galactose; GalNAc = N-acetylgalactosamine; Sia = sialic acid. D: σ_A_ weighted F_o_-F_c_ omit map of the GT1b analog contoured at 2.3 σ; oriented approximately as in C.

Gangliosides have been shown to be critical for the toxicity and binding of BoNT serotypes A, B and G, for which the protein receptors have also been identified. When ganglioside biosynthesis is inhibited in neuroblastoma cells, BoNT/A is inactive, likely because of its inability to penetrate the cells in the absence of gangliosides [12]. Gangliosides consist of a lipid part (a ceramide) linked to a complex polysaccharide head group displayed on the membrane surface. The polysaccharide groups contain sialic acids, but the number, composition and positions of the monosaccharide units vary between different gangliosides (Figure 2). GT1b is the ganglioside with the highest affinity to several of the toxin serotypes, including BoNT/A and B, and its carbohydrate moiety is composed of seven monosaccharides (Figure 2). All toxin serotypes, except D, utilize gangliosides as co-receptors [13],[14].

A dual-receptor model has been proposed for the infective process of BoNT, necessitating binding with both a protein receptor and a ganglioside co-receptor [11],[15] to induce paralysis. It has been suggested that the acidic environment of the endocytotically absorbed vesicle induces a drastic rearrangement of BoNT, specifically in the N-terminal part of its heavy chain, the translocation domain. This rearrangement leads to the translocation of the light chain into the cytosol, possibly through a transmembrane channel [2]. The extreme potency of the BoNTs and the high affinity to their targets is the result of the simultaneous interaction between the toxin and its two co-receptors on the cell surface (Figure 1).

**Figure 3:**
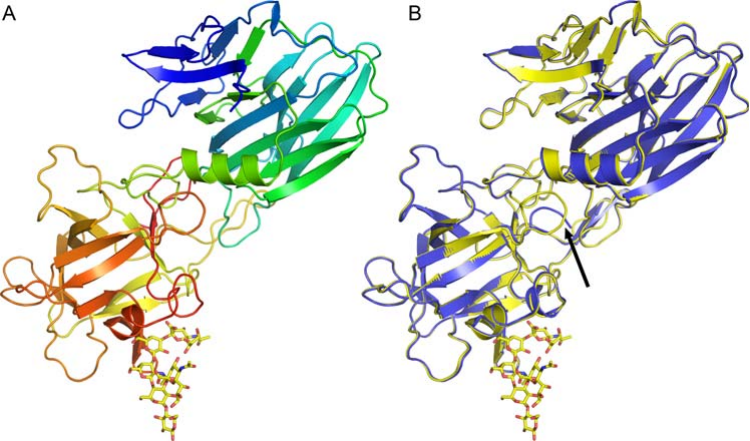
Binding domain of BoNT/A in complex with GT1b and comparison with the Apo structure. A: Overall ribbon representation of the BoNT/A binding domain in rainbow color representation, from the N-terminus (blue) to the C-terminus (red). The GT1b polysaccharide as yellow sticks. B: Comparison of the Apo structure (blue ribbons) and the GT1b complex (yellow ribbons). The GT1b polysaccharide is shown as yellow sticks. The position of the 1228–1234 loop is indicated by an arrow.

**Figure 4:**
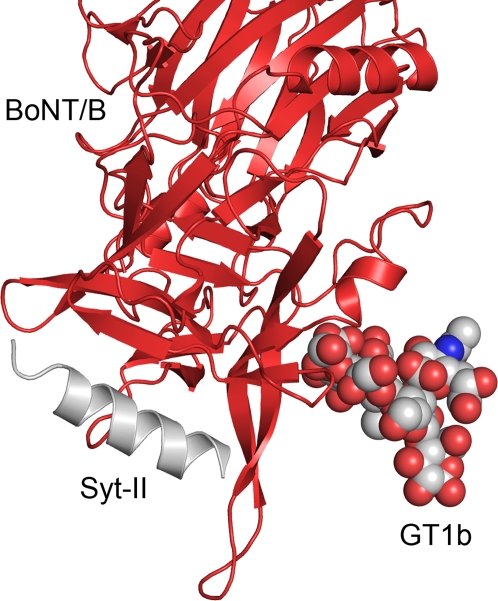
BoNT/B bound to Syt-II with the overlaid GT1b modeled from the BoNT/A binding domain complex. BoNT/B as red ribbon; Syt-II in grey ribbon and GT1b represented as CPK spheres.

We have solved the crystal structure of the binding domain of BoNT/A in complex with the polysaccharide moiety of the ganglioside GT1b (Figure 3A), the first step in cell recognition. The toxin interacts with one protein receptor, and also with a specific ganglioside co-receptor. The first structure of a BoNT (BoNT/B) in complex with its protein receptor (Synaptotagmin II; Syt-II) was previously reported [8],[9]. Now, we can present the first structure of a BoNT in complex with its ganglioside co-receptor, GT1b. By applying the information we have obtained about this ganglioside interaction, we can generate a picture of the toxin's simultaneous interaction with its two co-receptors (Figure 1 and Figure 4). The final model supports the model, where the long helixes of the translocation domain enter the cell membrane at a steep angle [16] Figure 1. We now believe that the high affinity generated by the interaction with the two co-receptors is simply a product of the two individual affinities, without any major contributions from allosteric effects induced by the ganglioside. We believe that the general features of the ganglioside binding observed here are representative for all the six ganglioside interacting BoNT serotypes (i.e. all serotypes except D).

## Materials and Methods

### GT1b analog synthesis

The GT1b analog was synthesized from the lactose derivative 2-(trimethylsilyl)ethyl 2,6-di-*O*-benzyl-β-D-galactopyranosyl-(1→4)-2,3,6-tri-*O*-benzyl-β-D-glucopyranoside using the reaction scheme described by Ishida *et al.* [17].

### Protein expression and purification

The C-terminal heavy chain of BoNT/A1, residues 876–1296, was cloned into a pET28a His-tag vector (Novagen). For protein expression, transformed BL21-AI *E. coli* cells were precultured in LB medium containing kanamycin (50 µg/ml) at 37°C overnight. The prepared pre-inoculum was transferred to twelve 1000 ml cultures of LB media containing 50 µg/ml kanamycin and incubated at 37°C until the OD at 600 nm reached 0.6. Protein expression was induced with 1 mM isopropyl β-D-1-thiogalactopyranoside (IPTG) and 0.2% arabinose, and the culture was incubated at 37°C for 4 hours. Cells were resuspended in a lysis buffer containing 50 mM Tris-HCl pH 8.0, 500 mM NaCl, 10 mM imidazole and EDTA-free protease inhibitors (Roche) followed by the addition of lysozyme (0.3 mg/ml). After 30 min incubation at room temperature, benzonase was added (0.5 U/µl), the cells were incubated 30 min at room temperature and the cells were sonicated. The crude lysate was clarified by centrifugation at 100 000*g* for 45 min at 5°C and filtration through a 0.45 µm membrane. The supernatant was loaded onto a column packed with Nickel IMAC Sepharose 6 Fast Flow resin (GE Healthcare) equilibrated with 20 mM Tris-HCl buffer pH 8.0 containing 500 mM NaCl and 10 mM imidazole. Elution was performed using 500 mM imidazole in the same buffer.

The protein sample was further purified by size-exclusion chromatography using a Sephacryl S-200 HR column (GE Healthcare) pre-equilibrated with 10 mM Tris-HCl buffer pH 8.0, 20 mM NaCl, 1 mM EDTA and 0.1% Triton X-100. Pure protein was concentrated using 30 kDa cutoff filters (Centricon). The protein concentration was determined by UV-Vis absorbance measurements using an extinction coefficient of 86 250 M^−1^ cm^−1^.

### Crystallization and structure determination

0.8 µl of the BoNT/A binding domain (15 mg/ml) in a buffer containing 6 mM GT1b analog, 13 mM Tris pH 8, 18 mM NaCl, 0.5 mM EDTA, 0.05% Triton-X-100 was mixed with 0.8 µl well solution (21% PEG3350, 0.2 M MgCl_2_, 0.1 M BisTris pH 5.5) in a hanging drop experiment at 20°C. The GT1b-protein solution was preincubated for 30 min prior to setting up the drops. For the apo structure, the protein concentration was 13 mg/ml, GT1b was omitted and the well solution was 18% PEG3350, 0.2 M MgCl_2_, 0.1 M BisTris pH 5.2. The crystals were flash frozen in liquid nitrogen after the addition of well solution complemented with 20% glycerol to the crystallization drop. Diffraction data was collected at 100 K on beamline 11.1 at the Stanford Synchrotron Radiation Laboratory (SSRL). The data was processed using the programs XDS and XSCALE [18]; statistics are presented in Table 1. The binding domain from the BoNT/A holotoxin (PDB: 2NYY) was used as a search model using MOLREP [19]. wARP [20] was used to build an initial model that was refined by iterative rounds of model building using Coot [21] and Refmac5 [22] with 10 TLS groups [23]. The final model of the GT1b complex includes BoNT/A residues 873–1297. The apo structure residues 1229 and 1230, and the CH_2_CH_2_Si(Me)_3_ and Sia7 of the GT1b analog in the complex were disordered and therefore not modeled. His 873–Asp 875 and Gln 1297 that are results of the cloning procedure are included in the final structures. Interestingly the additional C-terminal glutamine is making extensive crystal contacts; possibly being important for the formation of the crystal lattice. ProDRG [24] was used to generate the geometrical restraints for the GT1b analog. PyMol (www.pymol.org) was used to generate illustrations. ESPript [25] and SSM [26] was used for the structural alignments. Coordinates for both structures have been deposited in the Protein Data Bank. The accession numbers are: BoNT/A binding domain (2VUA), and BoNT/A binding domain-GT1b complex (2VU9).

**Table 1:** Data-processing and refinement statistics.

	BoNT/A binding domain Apo Structure	BoNT/A binding domain GT1b Analog Complex
Space group	C222_1_	C222_1_
Unit cell (a,b,c in Å)	73.0, 114.5, 105.8	72.7, 116.1, 105.5
Resolution (Å)	20-1.7 (1.8-1.7)	20-1.6 (1.7-1.6)
R_sym_ (%)	4.0 (53.1)	4.2 (40.5)
<I/σI>	27.0 (4.2)	27.1 (5.3)
Completeness (%)	99.0 (98.4)	99.6 (99.2)
Redundancy	7.8 (7.5)	7.4 (7.3)
R_cryst_ (%)	17.0	16.2
R_free_ (%)	20.5	18.6
r.m.s. deviation bond length (Å)	0.014	0.012
r.m.s. deviation bond angle (°)	1.5	1.5
**Average B factors (Å^2^)**		
Protein	24	16
Solvent	42	35
GT1b		36
**Ramachandran plot**		
Most favored (%)	87.4	88.2
Additional allowed (%)	12.1	11.3
Generously allowed (%)	0.3	0.3
Disallowed (%)*	0.3	0.3

*: Two ramachandran plot outliers Asn 1025 and Asn 1127 are clearly defined by the electron density, Asn1127 is involved in a strong crystal packing interaction.

### Accession numbers

The Protein Data Bank (http://www.rcsb.org/pdb) accession numbers for the coordinates for the structures of the complexes presented in this article; apo BoNT/A binding domain (2VUA), and BoNT/A binding domain-GT1b complex (2VU9). The Protein Data Bank accession numbers for the additional structures discussed in this paper are; BoNT/A holotoxin, 3BTA; BoNT/A holotoxin (2.6 Å), 2NYY; BoNT/B holotoxin, 1EPW; BoNT/B with trisaccharide, 1F31; BoNT/B with Syt-II, 2NP0 and 2NM1; BoNT/B binding domain, 1Z0H; TeNT binding domain with ganglioside, 1FV2; Siglec-7 with GT1b, 2HRL and Cholera toxin with GM1, 2CHB. Swiss-Prot accession numbers; BoNT/D, P19321 and BoNT/G, Q60393.

## Results

Here, we report the crystal structure of the BoNT/A binding domain to 1.7 Å, and its complex with the polysaccharide moiety of GT1b to 1.6 Å. Three BoNT/A holotoxin structures have been reported previously [5],[27]; the highest resolution structure was that of the BoNT/A holotoxin complexed with a monoclonal antibody to 2.6 Å [27]. The BoNT/A binding domain has two sub domains that each consist mainly of β–sheets (Figure 3A). The N-terminal half has an all β-sheet, jelly roll barrel fold, while the C-terminal half has a β-trefoil fold. Clear density is observed for the key shallow grove binding pocket anchored by Trp 1266.

### GT1b–BoNT/A binding domain complex

In total, eight BoNT residues make hydrogen bonds to the GT1b polysaccharide, five of the hydrogen bonds are to Gal4 and GalNAc3 (Figure 2C) that also have the lowest B values of the GT1b monosaccharides. Because of reports of a slow conformational change being induced in BoNT/A by GT1b binding [28], we pre-incubated the protein with the GT1b analog at pH 8 in a low ionic strength buffer for approximately 30 min prior to setting up crystallization trials. The GT1b complex crystals grew at pH 5.5 which is comparable to the pH inside the endosome; this indicates that the neurotoxin ganglioside complex could be stable also inside the endosome. Six of the seven monosaccharides in the GT1b analog are clearly defined by the electron density, while Sia7 is disordered (Figure 2D). The ganglioside GD1a is distinguished from GT1b only by the absence of the Sia7 moiety, yet GD1a still displays high affinity for the toxin [28]. The GT1b analog used here differs from GT1b only in the replacement of the ceramide (the lipid), that would be buried inside the membrane, for a 2-(trimethylsilyl)ethyl group (Figure 2B).

**Figure 5:**
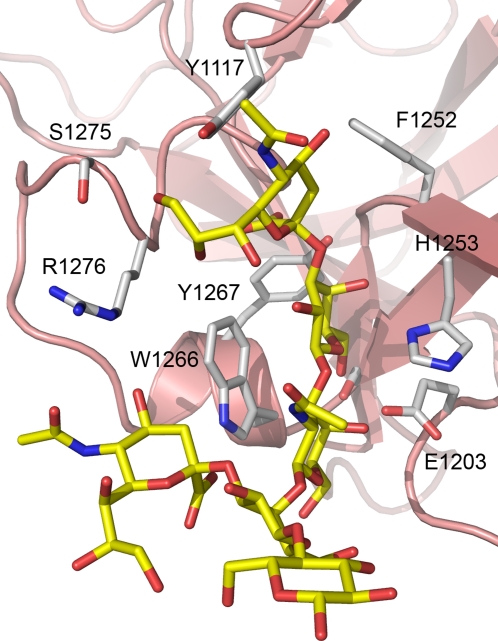
Close-up of the GT1b binding site. GT1b represented as sticks with yellow carbons. The GT1b coordinating residues are shown as sticks with grey carbons.

Trp 1266 is conserved among the BoNT serotypes, and on BoNT/A is located in a binding groove that makes extensive interactions with GT1b. Gal4 and GalNAc3 interact with Trp 1266 through hydrophobic stacking (Figure 2C and Figure 5). Additionally, the indole nitrogen of Trp 1266 hydrogen bonds with the carboxylic acid group of Sia6 (3.1 Å); further underlining the importance of Trp 1266 in ganglioside binding (Figure 2C). Tyr 1267 is also conserved in all ganglioside binding BoNT serotypes [29], and extends the hydrophobic part of the binding pocket generated by Trp 1266 (Figure 2C and Figure 5).

The interactions between GT1b and the toxin are shown in Figure 2C and a table describing the interactions is available as Table S1. In addition, some of the most important interactions are described here. Ser 1264 (2.7 Å) and His 1253 (2.8 Å) hydrogen bonds to Gal4, both of these residues are highly conserved between the different serotypes and also to the TeNT. Glu 1203 hydrogen bonds to both Gal4 (2.7 Å) and GalNAc3 (2.6 Å) and the carbonyl oxygen of Phe 1252 coordinate Gal4 (2.6 Å) (Figure 2C). There are also two internal hydrogen bonds within GT1b.

The importance of several of the residues in BoNT/A involved in GT1b binding has previously been identified by mutational studies [29]. All mutants that lower the affinity to GT1b are targeting residues that make direct interactions with the GT1b in the complex. Importantly, mutants of Trp 1266 and Tyr 1267 fail to bind GT1b; in fact, even the removal of a single oxygen atom from the binding site, as in the Y1267F mutant, leads to a dramatically lower affinity for GT1b [29]. The two hydrogen bonds formed by the hydroxyl group of Tyr 1267 to the backbone of Phe 1252 are likely to be important for the correct positioning of tyrosine and the structural integrity of the binding site. Other residues where the interaction is dramatically affected by mutation (His 1253, Glu 1203, Ser 1264) are making crucial hydrogen bonds to GT1b [29].

Four water molecules mediate hydrogen bonds between BoNT/A and GT1b; additionally, three water molecules mediate internal hydrogen bonds within GT1b; these are all listed in Table S1. All of these water molecules are likely to be important for the binding of GT1b to the toxin. Two of the water molecules that are mediating hydrogen bonds between GT1b and the protein (3376 and 3350) have counterparts located in the same positions in the Apo structure; interestingly water 3350 also has a counterpart in the BoNT/B structures [6],[8],[9]. This emphasizes the fact that the ganglioside binding site is preset for binding and few structural changes are necessary for optimal binding to gangliosides.

### Apo BoNT/A binding domain structure

We have also solved the structure of the BoNT/A binding domain in the absence of ganglioside. Circular dichroism (CD) measurements on BoNT/A have indicated that there are large conformational changes induced by GT1b binding, leading to an increase in the helical contents and a decrease to less than half of the β-sheet contents [28]. The GT1b complex structure and the apo structure reported here are very similar with an overall rmsd of 0.3 Å (Figure 3B). It is possible that BoNT/A holotoxin become partially inserted into the low dielectric environment of the GT1b micelles. This insertion would dramatically influence the CD spectra. The GT1b analog complex reported here represents the toxin bound to the presynaptic membrane before the translocation process is initiated.

Some structural differences are observed between the GT1b complex and the apo structure. The side chain of Arg 1276 has moved 1 Å closer to the position of Sia6 and the side chain of Trp 1266 has rotated 9 degrees, moving it closer to the hydrophobic face of Gal4. But most of the amino acids directly interacting with GT1b have similar conformations in the two structures. The exception is Tyr 1117, which directly coordinates GT1b. Tyr 1117 has rotated 25 degrees around the C-beta C-gamma bond upon the binding of GT1b; in addition to this rotation the hydroxyl group of Tyr 1117 has moved 1.2 Å to a position where it can form two hydrogen bonds to Sia5 (Figure 2C). Beyond the major area of interaction, one region of the protein that has major differences between the GT1b complex and the Apo structure is the 1228–1234 loop which has adopted an alternative conformation. The C-alpha of residue 1231 has moved as much as 8 Å, and residues 1229 and 1230 of this loop are disordered in the Apo structure but clearly visible in the GT1b complex (Figure 3B). The distance between GT1b and the 1228–1234 loop is approximately 15 Å, while the side chains of Leu 1116, sitting next to Tyr 1117, and Ile 1231 located on the 1228–1234 loop are 4 Å apart. There is a slight shift in the positions of Leu 1116 and Tyr 1117 between the structures which opens for the possibility that the conformational change of the 1228–1234 loop is induced by GT1b binding; but it is more likely that this change is instead induced by a small shift in the crystal packing.

### Comparison of BoNT/A holotoxin and binding domain structure

The 1230 loop region in the holotoxin structure (residues 1226–1236) [5],[27] points away from binding domain relative to the apo structure, specifically Gly 1230 that moves 12 Å. In addition, residues 1271–1277 have also reoriented when comparing the BoNT/A holotoxin structure. Ser 1275 and Arg 1276 that coordinate Sia5 in the GT1b complex are located within this section. However, the positions of residues 1271–1277 are nearly identical when comparing the GT1b complex and apo binding domain structure presented here. Given the location of this region and how the molecules pack in the crystal lattice when comparing the holotoxin structure and the binding domain, it is possible that these changes are caused by the differences in crystal packing. Another segment of the binding domain facing the translocation domain in the holotoxin structure (928–939) has adopted an alternative conformation with C-alpha positions moving by up to 10 Å. This is likely to be the result of the exposure of the hydrophobic translocation domain interaction area to solvent. A rearrangement of approximately the same section of the binding domain of BoNT/B has been reported when the binding domain is detached from the rest of the toxin [30]; this segment in the BoNT/A and BoNT/B binding domains have different orientations. The regions where changes have occurred in BoNT/A are highlighted in the structure based sequence alignment available as Figure S1.

## Discussion

BoNT/A and BoNT/B have a single ganglioside binding site [29], Trp 1266 of BoNT/A and residues in its proximity have been shown by several investigators to be critical for ganglioside binding; mutations of residues in this region abolish ganglioside binding [29]. Ganglioside binding quenches the tryptophan fluorescence of BoNT/A and the only solvent exposed tryptophan in BoNT/A is Trp 1266 [31],[32]. Even though many reports have highlighted the importance of gangliosides and the Trp 1266 binding region, the details of the interactions have been elusive. Studies of trypsin digested BoNT/A have indicated that the last 30 amino acids of the toxin are important for toxicity [33]. Structural data on the binding of sialyllactose to BoNT/B [6] have indicated that there could be large differences in ganglioside binding between the TeNT [34] and the BoNTs. Also, it has recently been suggested that the Syt-II binding site in BoNT/B would be in direct contact with the Sia5 moiety of GT1b [8]; this would place Sia5 approximately 20 Å from the Sia5 position observed in the Hc/A-GT1b complex presented here.

Generally, the ganglioside is believed to bind the toxin without inducing any large conformational changes, much as in the lock and key model (Figure 3B); but there have been several suggestions in the literature that gangliosides change the affinity between the toxin and its protein receptor; either by inducing conformational changes and/or by direct interactions. GT1b binds to all serotypes of BoNT that have been shown to interact with gangliosides, but the ganglioside specificity varies between serotypes. GT1b is the ganglioside with highest affinity for serotype A and B. Serotypes A, B, C, E and F all bind GT1b and GD1a. Serotype A, B, C and F also bind GD1b (Figure 2A). The ganglioside GQ1b can bind serotype A and E [13]. Serotype D has been reported not to bind or interact with gangliosides, but instead BoNT/D interacts with phosphatidylethanolamine [14]. Serotype G interacts with gangliosides but the specificity is unclear [11],[35]. While most BoNTs have been shown to interact with gangliosides, the specificities above should be interpreted cautiously; since all serotypes have not been tested with all the different gangliosides.

We now suggest that the toxins interactions with its receptors are not as complex as previously believed. Only the simultaneous interactions with two receptors are necessary to obtain the high avidity by the “dual receptor” model. The existence of a major communication between these two binding sites might be a more complex mechanism than needed to explain the interaction. The polysaccharide head group of GT1b is dynamic, and we have now shown that the ganglioside binding site of BoNT/A is rigid with very limited structural changes being induced upon binding. Binding to a static binding site is not a general mechanism for ganglioside interactions since conformational changes are induced by the binding of GT1b to the Siglec-7 receptor. Siglec-7 is involved in signaling in the immune and nervous systems [36], where specificity and control are likely to be important factors for binding. There are some similarities in the GT1b interactions between Siglec-7 and BoNT/A; they both have shallow binding pockets with tryptophans involved in hydrophobic stacking interactions. The structure of cholera toxin in complex with the ganglioside GM1 also reveals similarities [37], a shallow binding groove binds the ganglioside and a key tryptophan makes hydrophobic interactions. In all of these complexes the gangliosides are positioned by several hydrogen bonds in addition to tryptophan interactions. The tryptophan interaction appears to be a common hallmark of the interactions between gangliosides and proteins.

### Comparison between the TeNT GT1b analog complex [34] and the BoNT/A GT1b analog complex

**Figure 6:**
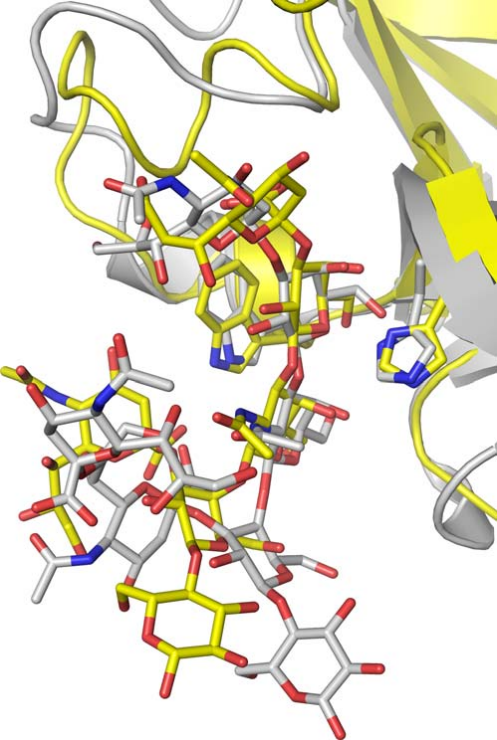
A comparison of a GT1b β-anomer (gray sticks) bound to the TeNT (gray ribbon), and GT1b (yellow sticks) bound to the BoNT/A binding domain (yellow ribbon). The BoNT/A binding domain residues Trp 1266 and His 1253 and corresponding TeNT residues Trp 1289 and His 1271 are shown as sticks.

We can now show that BoNT/A binds to gangliosides in the same binding site and in a similar manner to TeNT (Figure 6), though there are some large differences in the interaction. Gal4 and GalNac3 are bound in a similar manner and make key interactions with, conserved or semi-conserved residues. The Sia5 group is critical for the affinity of BoNT/A to gangliosides, since the affinity to GD1a is much higher than that of GM1 [28]. The TeNT has a high affinity for GT1b and GQ1b, but also for GD1b [38],[39], which is GT1b without the Sia5 unit (Figure 2A). There is approximately 2 Å difference between the positions of the Sia5 group in the TeNT complex and the BoNT/A complex structures (Figure 6). Tyr 1117 and Phe 1252 in BoNT/A make hydrophobic interactions with Sia5, and the presence of these two bulky residues leads to a more closed Sia5 binding site in BoNT/A versus TeNT complex structure where these interactions among the comparable residues Ala 1134 and Thr 1270 are absent (Figure 6). Interestingly, Ser 1275 of BoNT/A, which makes a bond to Sia5, is not conserved.

Gal4 and GalNAc3 are coordinated by Glu 1203 in the BoNT/A complex; in the TeNT complex structure Asp 1222 is present in this position [34]. The carboxyl group of Asp 1222 in the TeNT complex is orientated in a different way but coordinates the same positions on Gal4 and GalNAc3 as Glu 1203 in the BoNT/A complex (Figure 2C). Glu 1203 is conserved among all ganglioside binding BoNT serotypes except serotype G where it is replaced by a glutamine [29], which could be able to coordinate the ganglioside in a similar way. TeNT has an additional ganglioside binding site which corresponds to the region around Arg 1131 in BoNT/A [34],[40]; but this area of BoNT/A has large structural differences from TeNT and does not participate in ganglioside binding in BoNT/A. This is in agreement with the finding that BoNT/A and BoNT/B have a single ganglioside binding site [29]. GT1b in the BoNT/A complex does makes two interactions with a crystallographically related molecule; Glu 969 and Asn 970 interact with Sia6; although, these residues are not conserved and the interaction is unlikely to have any biological significance.

Another difference between the TeNT ganglioside complex and the BoNT/A GT1b analog complex includes the orientation of the disialic arm (Sia6) which is very different between the two structures, and the Glc1–Gal2 saccharides are in a different position (Figure 6). This is not surprising since the disialic arm (Sia6) is located in a different position and there are no interactions between the Glc1–Gal2 saccharides and the protein. A non natural β-anomer of Sia6 is present in the GT1b analog used in the TeNT complex [34], while we use the natural α-anomer. This difference changes the relative positions of the Sia6 ring and the Sia6 carboxylic acid group. Trp 1266 in the BoNT/A structure coordinates the carboxylic acid group of Sia6, the corresponding residue in TeNT is Trp 1289 (Figure 6). It is unclear how GT1b would interact with the second GT1b binding site of TeNT and Trp 1289 if the α-anomer of GT1b were used. It is likely that the general features of the TeNT GT1b binding would be unaltered, but that the position and interactions of the Sia6 and its carboxylic acid group with the protein would change.

### Co-receptor binding to serotype B

With the structure of the complex between BoNT/B and its protein receptor Syt-II that has been published [8],[9], we can now combine our structural data of BoNT/A with its ganglioside co-receptor to produce a model of the “double receptor” interaction proposed by Montecucco *et al.* [15]. Additionally, we can observe that GT1b in our complex with BoNT/A binds to the same binding pocket as the trisaccharide sialyllactose in the complex with BoNT/B that has also has been determined [6]. However, there are very large differences in binding. The Sia unit of sialyllactose in the BoNT/B complex is rotated by 180 degrees compared with the Sia5 of the BoNT/A GT1b complex and located approximately where Gal4 is located in the BoNT/A GT1b structure. The trisaccharide sialyllactose is only a partial mimic of a ganglioside, and would be unable to make the key interactions we observe in the GT1b complex. We believe that the sialyllactose complex has correctly identified the ganglioside binding pocket of BoNT/B, but that the binding of a ganglioside to BoNT/B would be similar to the binding of GT1b to BoNT/A.

The key residues in coordinating the GalNAc3–Gal4 part of GT1b are all conserved between the BoNT/A and BoNT/B. Trp 1266, Glu1203, His 1253, Ser 1264 in BoNT/A correspond to Trp 1261, His 1240, Glu 1189 and Ser 1259 in BoNT/B. Phe 1252 in BoNT/A is not conserved in BoNT/B, but the position of the carbonyl oxygen coordinating GT1b is similar. The main differences are in the coordination of Sia5; Tyr 1117, Phe 1252 and Ser 1275 that interact with Sia5 in our BoNT/A complex are not conserved in BoNT/B. The Sia5 of GT1b has been reported to be important for the interaction between Syt-I and BoNT/B. This could be the result of the simultaneous binding to gangliosides and Syt constructs present in the same micelles rather than a direct interaction between the co-receptors [11]. Our studies on BoNT/A cannot completely rule out conformational changes in BoNT/B induced by ganglioside binding leading to an increased affinity for Syt-I.

The residues that are important for GT1b binding in BoNT/A have also been mutated in BoNT/B; and the results are very similar for both serotypes [29]. Since the binding of the GalNAc3–Gal4 part of GT1b is similar between TeNT complex and our complex and the key residues in coordinating GalNAc3–Gal4 are conserved between BoNT/A and B. We believe that GT1b binding to BoNT/B will be similar to the binding to BoNT/A. Furthermore, it is likely that the ganglioside binding of BoNT/A is representative of all ganglioside interacting BoNT serotypes. The conserved residues corresponding to Trp 1266, Tyr 1267 and Gly 1290 in BoNT/A have been mutated in BoNT/C and are also important for ganglioside binding in serotype C [41]. These residues are all part of the ganglioside binding pocket of the BoNT/A GT1b complex presented here.

### Implications for the “double receptor” model

In the GT1b polysaccharide complex presented here, the distance between the position where the ceramide of GT1b would be located (Figure 2) and the toxin is 15 Å. This gives an approximate value of the spacing between BoNT/A and the presynaptic membrane, and puts some restrictions on the toxins interactions with the different protein receptors of the BoNT serotypes. The binding site on the toxin has to be able to access the epitope on the protein receptor while being attached to the presynaptic membrane by the ganglioside. This holds true for a modeled ternary complex of BoNT/B, where these distances are in perfect agreement. It is very likely the initial contact between the toxin and the neuronal membrane is mediated by GT1b; this increases the local toxin concentration at the membrane surface dramatically and gives the toxin the opportunity to diffuse in the plane of the membrane and bind its protein receptor. When GT1b is modeled into the Syt-II complex of BoNT/B [8], based on its binding to the BoNT/A binding domain, the two points of attachment to the presynaptic membrane are 22 Å apart (Figure 4). This model gives us the most detailed view yet on the nature of the “double receptor” model of a BoNT. In this model, BoNT/B can rotate around the axis formed by the two tethers to the membrane, but this model puts rather strict restraints on the binding, elucidating a well-defined view of the interaction (Figure 1 and Figure 4). We now have structural data for several of the stages of the toxins binding to the neuronal surface; this is presented in Figure 1. There is a long extended loop (the 1250 loop) pointing out from BoNT/B located between the Syt-II binding site and the ganglioside binding site (Figure 4). This loop has a very hydrophobic tip, consisting of Gly 1246, Ile 1247, Val 1248 and Phe 1249. These residues are completely exposed on the surface, and it is possible that they play a part in BoNT/B binding to the membrane. The axis of possible rotation is perpendicular to the 100 Å long helixes of the translocation domain (Figure 1 and Figure 4). The rotation around this two-point attachment hinge makes it possible for one end of the long helix bundle of the translocation domain to directly interact with the membrane, supporting a translocation domain insertion model previously suggested [8],[16]. The N-terminal (loops 600 and 760 in BoNT/A and 590 and 750 in BoNT/B) end of the translocation domain has better sterical access to the membrane surface and is closer to the cluster of negative charges and the histidines that have been suggested as key residues for the insertion of the translocation domain into the membrane; but additional experiments are necessary to clarify which end of the translocation domain makes the initial contact with the membrane [32]. The model presented here gives us a glimpse at the initiation of toxin translocation (Figure 1 and Figure 4).

The interaction of BoNT/A and gangliosides, including GT1b, is critical for the toxins passage over the digestive epithelial barrier [42]. Additionally, it is likely that SV2, or an as yet unidentified receptor, together with gangliosides is necessary for absorption [42]. In a recent study, the non-toxic neurotoxin-associated proteins (NAPs) did not influence the toxins passage over the epithelial barrier; similar results were obtained with toxin alone and with the toxin-NAP complex [42]. The role of the NAPs is under debate, but it is possible that their major function is to protect the toxin from proteases and the acidic environment of the stomach and not in absorbance. In a competition experiment, where the toxin was pre incubated with GT1b, a large decrease in the absorption of the toxin was observed [42]. This would suggest that the GT1b binding site of the neurotoxin itself is responsible for the interaction with the epithelial cells and the absorption of the toxin, and not the NAPs as previously thought. These results further highlight the important role of the neurotoxin's GT1b binding site. Inhibitors targeting GT1b binding could disrupt both the absorption from the digestive tract and the passage into the nerve cells.

## Supporting Information

Figure S1 (0.19 MB DOC)

Table S1 (0.05 MB DOC)

## References

[1] Breidenbach MA, Brunger AT (2005). New insights into clostridial neurotoxin-SNARE interactions.. Trends Mol Med.

[2] Turton K, Chaddock JA, Acharya KR (2002). Botulinum and tetanus neurotoxins: structure, function and therapeutic utility.. Trends Biochem Sci.

[3] Shukla HD, Sharma SK (2005). Clostridium botulinum: A bug with beauty and weapon.. Crit Rev Microbiol.

[4] Bhidayasiri R, Truong DD (2005). Expanding use of botulinum toxin.. J Neurol Sci.

[5] Lacy DB, Tepp W, Cohen AC, DasGupta BR, Stevens RC (1998). Crystal structure of botulinum neuro-toxin type A and implications for toxicity.. Nat Struct Biol.

[6] Swaminathan S, Eswaramoorthy S (2000). Structural analysis of the catalytic and binding sites of Clostridium botulinum neurotoxin B.. Nat Struct Biol.

[7] Dong M, Yeh F, Tepp WH, Dean C, Johnson EA (2006). SV2 is the protein receptor for botulinum neurotoxin A.. Science.

[8] Chai Q, Arndt JW, Dong M, Tepp WH, Johnson EA (2006). Structural basis of cell surface receptor recognition by botulinum neurotoxin B.. Nature.

[9] Jin RS, Rummel A, Binz T, Brunger AT (2006). Botulinum neurotoxin B recognizes its protein receptor with high affinity and specificity.. Nature.

[10] Rummel A, Karnath T, Henke T, Bigalke H, Binz T (2004). Synaptotagmins I and II act as nerve cell receptors for botulinum neurotoxin G.. J Biol Chem.

[11] Rummel A, Eichner T, Weil T, Karnath T, Gutcaits A (2007). Identification of the protein receptor binding site of botulinum neurotoxins B and G proves the double-receptor concept.. Proc Natl Acad Sci U S A.

[12] Yowler BC, Kensinger RD, Schengrund CL (2002). Botulinum neurotoxin A activity is dependent upon the presence of specific gangliosides in neuroblastoma cells expressing synaptotagmin I.. J Biol Chem.

[13] Yowler BC, Schengrund CL (2004). Glycosphingolipids-Sweets for botulinum neurotoxin.. Glycoconjugate J.

[14] Tsukamoto K, Kohda T, Mukamoto M, Takeuchi K, Ihara H (2005). Binding of Clostridium botulinum type C and D neurotoxins to ganglioside and phospholipid. Novel insights into the receptor for clostridial neurotoxins.. J Biol Chem.

[15] Montecucco C (1986). How Do Tetanus and Botulinum Toxins Bind to Neuronal Membranes.. Trends Biochem Sci.

[16] Baldwin MR, Kim JJ, Barbieri JT (2007). Botulinum neurotoxin B-host receptor recognition: it takes two receptors to tango.. Nat Struct Mol Biol.

[17] Ishida HK, Ohta Y, Tsukada Y, Kiso M, Hasegawa A (1993). A synthetic approach to polysialogangliosides containing alpha-sialyl-(2–>8)-sialic acid: total synthesis of ganglioside GD3.. Carbohydr Res.

[18] Kabsch W (1993). Automatic Processing of Rotation Diffraction Data from Crystals of Initially Unknown Symmetry and Cell Constants.. J Appl Crystallogr.

[19] Vagin A, Teplyakov A (1997). MOLREP: an automated program for molecular replacement.. J Appl Crystallogr.

[20] Perrakis A, Morris R, Lamzin VS (1999). Automated protein model building combined with iterative structure refinement.. Nat Struct Biol.

[21] Emsley P, Cowtan K (2004). Coot: model-building tools for molecular graphics.. Acta Crystallogr D-Biol Crystallogr.

[22] Murshudov GN, Vagin AA, Dodson EJ (1997). Refinement of macromolecular structures by the maximum-likelihood method.. Acta Crystallogr D-Biol Crystallogr.

[23] Winn MD, Isupov MN, Murshudov GN (2001). Use of TLS parameters to model anisotropic displacements in macromolecular refinement.. Acta Crystallogr D-Biol Crystallogr.

[24] Schuttelkopf AW, van Aalten DM (2004). PRODRG: a tool for high-throughput crystallography of protein-ligand complexes.. Acta Crystallogr D-Biol Crystallogr.

[25] Gouet P, Robert X, Courcelle E (2003). ESPript/ENDscript: Extracting and rendering sequence and 3D information from atomic structures of proteins.. Nucleic Acids Res.

[26] Krissinel E, Henrick K (2004). Secondary-structure matching (SSM), a new tool for fast protein structure alignment in three dimensions.. Acta Crystallogr D-Biol Crystallogr.

[27] Garcia-Rodriguez C, Levy R, Arndt JW, Forsyth CM, Razai A (2007). Molecular evolution of antibody cross-reactivity for two subtypes of type A botulinum neurotoxin.. Nat Biotechnol.

[28] Yowler BC, Schengrund CL (2004). Botulinum neurotoxin a changes conformation upon binding to ganglioside GT1b.. Biochemistry.

[29] Rummel A, Mahrhold S, Bigalke H, Binz T (2004). The H-CC-domain of botulinum neurotoxins A and B exhibits a singular ganglioside binding site displaying serotype specific carbohydrate interaction.. Mol Microbiol.

[30] Jayaraman S, Eswaramoorthy S, Ahmed SA, Smith LA, Swaminathan S (2005). N-terminal helix reorients in recombinant C-fragment of Clostridium botulinum type B.. Biochem Biophys Res Commun.

[31] Kamata Y, Yoshimoto M, Kozaki S (1997). Interaction between botulinum neurotoxin type A and ganglioside: ganglioside inactivates the neurotoxin and quenches its tryptophan fluorescence.. Toxicon.

[32] Lacy DB, Stevens RC (1999). Sequence homology and structural analysis of the clostridial neurotoxins.. J Mol Biol.

[33] Shone CC, Hambleton P, Melling J (1985). Inactivation of Clostridium botulinum type A neurotoxin by trypsin and purification of two tryptic fragments. Proteolytic action near the COOH-terminus of the heavy subunit destroys toxin-binding activity.. Eur J Biochem.

[34] Fotinou C, Emsley P, Black I, Ando H, Ishida H (2001). The crystal structure of tetanus toxin Hc fragment complexed with a synthetic GT1b analogue suggests cross-linking between ganglioside receptors and the toxin.. J Biol Chem.

[35] Dong M, Tepp WH, Liu H, Johnson EA, Chapman ER (2007). Mechanism of botulinum neurotoxin B and G entry into hippocampal neurons.. J Cell Biol.

[36] Attrill H, Imamura A, Sharma RS, Kiso M, Crocker PR (2006). Siglec-7 undergoes a major conformational change when complexed with the alpha(2,8)-disialylganglioside GT1b.. J Biol Chem.

[37] Merritt EA, Sarfaty S, van den Akker F, L'Hoir C, Martial JA (1994). Crystal structure of cholera toxin B-pentamer bound to receptor GM1 pentasaccharide.. Protein Sci.

[38] Angstrom J, Teneberg S, Karlsson KA (1994). Delineation and Comparison of Ganglioside-Binding Epitopes for the Toxins of Vibrio-cholerae, Escherichia-coli, and Clostridium-tetani-Evidence for Overlapping Epitopes.. Proc Natl Acad Sci U S A.

[39] Holmgren J, Elwing H, Fredman P, Svennerholm L (1980). Polystyrene-Adsorbed Gangliosides for Investigation of the Structure of the Tetanus-Toxin Receptor.. Eur J Biochem.

[40] Rummel A, Bade S, Alves J, Bigalke H, Binz T (2003). Two carbohydrate binding sites in the H-cc-domain of tetanus neurotoxin are required for toxicity.. J Mol Biol.

[41] Tsukamoto K, Kozai Y, Ihara H, Kohda T, Mukamoto M (2008). Identification of the receptor-binding sites in the carboxyl-terminal half of the heavy chain of botulinum neurotoxin types C and D.. Microb Pathog.

[42] Couesnon A, Pereira Y, Popoff MR (2008). Receptor-mediated transcytosis of botulinum neurotoxin A through intestinal cell monolayers.. Cell Microbiol.

