# Supplementary material for: Crystal Structure of Botulinum Neurotoxin Type A in Complex with the Cell Surface Co-Receptor GT1b—Insight into the Toxin–Neuron Interaction
Source: PLoS Pathog. 2008 Aug 15;4(8):e1000129. doi: 10.1371/journal.ppat.1000129 (PMC2493045; doi:10.1371/journal.ppat.1000129)

**Supporting Figure S1:**

A structure-based sequence alignment including the binding domains of BoNT/A (HcA; this work), BoNT/B (HcB; PDB: 1Z0H) and TeNT (HcT; PDB: 1FV2). The residues making hydrogen bonds to GT1b in BoNT/A are marked by stars. The two regions where structural changes are observed in BoNT/A are marked with triangles. The numbering refers to the BoNT/A structure. ESPript [1] and SSM [2] were used to generate this figure.

1. Gouet P, Robert X, Courcelle E (2003) ESPript/ENDscript: Extracting and rendering sequence and 3D information from atomic structures of proteins. Nucleic Acids Res 31: 3320-3323.

2. Krissinel E, Henrick K (2004) Secondary-structure matching (SSM), a new tool for fast protein structure alignment in three dimensions. Acta Crystallogr D Biol Crystallogr 60: 2256-2268.


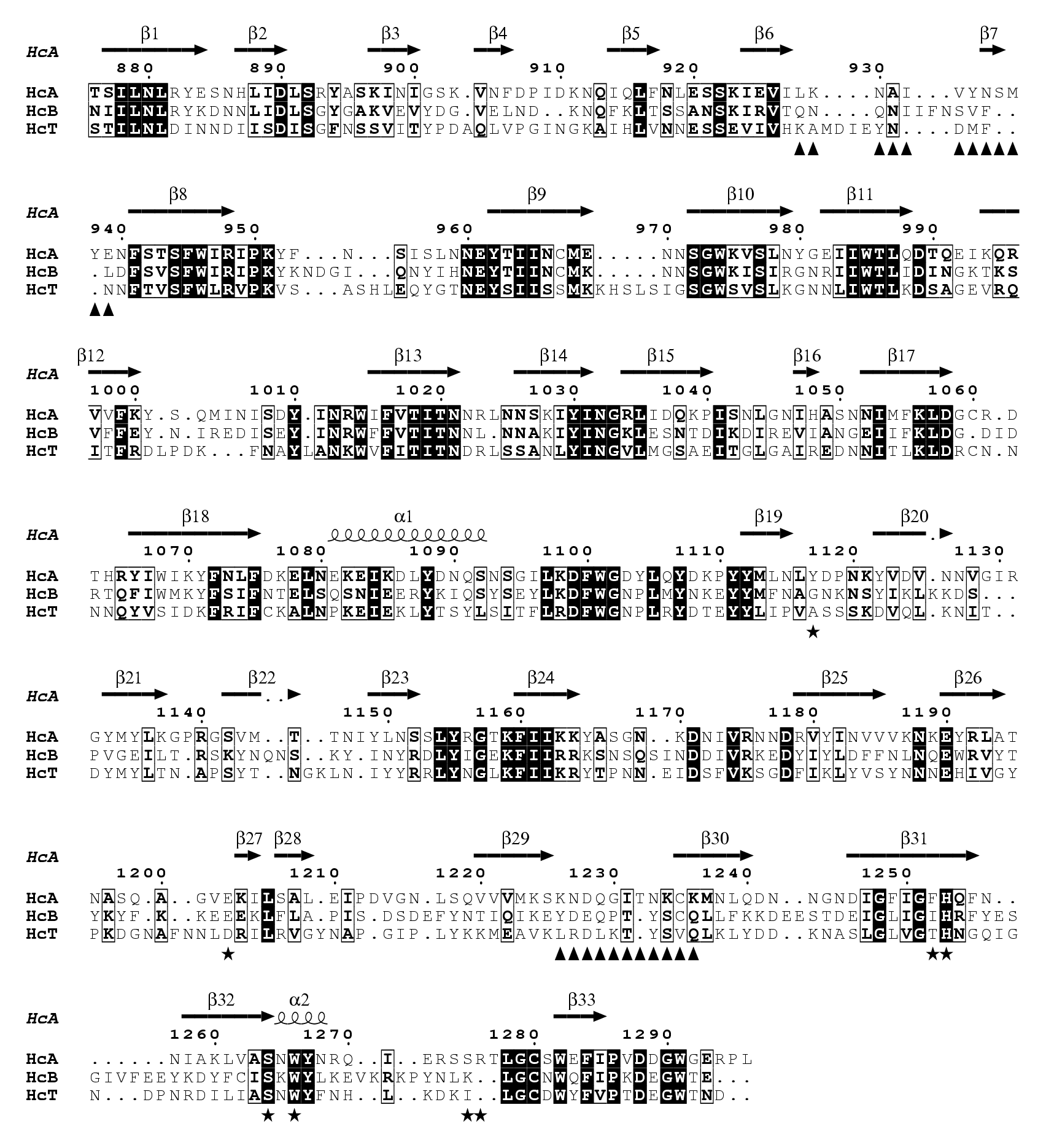

Supplement: Figure S1 — (0.19 MB DOC) [file ppat.1000129.s001.doc]
