# Supplementary material for: Crystal Structure of Botulinum Neurotoxin Type A in Complex with the Cell Surface Co-Receptor GT1b—Insight into the Toxin–Neuron Interaction
Source: PLoS Pathog. 2008 Aug 15;4(8):e1000129. doi: 10.1371/journal.ppat.1000129 (PMC2493045; doi:10.1371/journal.ppat.1000129)
Supplement: Table S1 — (0.05 MB DOC) [file ppat.1000129.s002.doc]

**Supplementary tables S1:**

**Hydrogen bonds between the toxin and GT1b**

| **Protein atom** | **Distance (Å)** | **GT1b sugar atom** |
| --- | --- | --- |
| Tyr 1117 (OH) | 2.8 | Sia5 (N5) |
| Tyr 1117 (OH) | 3.0 | Sia5 (O9) |
| Glu 1203 (OE2) | 2.7 | Gal4 (O6) |
| Glu 1203 (OE2) | 2.6 | GalNAc3 (O4) |
| Phe 1252 (O) | 2.6 | Gal4 (O4) |
| His 1253 (ND1) | 2.8 | Gal4 (O4) |
| Ser 1264 (OG) | 2.7 | Gal4 (O6) |
| Trp 1266 (NE1) | 3.1 | Sia6 (O1B) |
| Ser 1275 (OG) | 3.2 | Sia5 (O9) |
| Arg 1276 (NE) | 3.1 | Sia6 (O4) |

**Interactions between toxin and GT1b bridged by water**

| **Protein atom** | **Distance (Å)** | **Bridging Water** | **Distance (Å)** | **GT1b sugar atom** |
| --- | --- | --- | --- | --- |
| Gly 1279 (N) | 2.7 | 3376 | 2.7 | Sia5 (O1B) |
| Arg 1276 (O) | 3.1 | 3376 | 2.7 | Sia5 (O1B) |
| Arg 1269 (NH1) | 2.9 | 3362 | 3.1 | GalNAc3 (O6) |
| Gln 1254 (N) | 3.1 | 3350 | 3.1 | Gal4 (O3) |
| Gln 1270 (OE1) | 2.6 | 3396 | 2.5 | Sia6 (O4) |

**GT1b internal hydrogen** bonds

| **GT1b sugar atom** | **Distance (Å)** | **GT1b sugar atom** |
| --- | --- | --- |
| Sia5 (O7) | 3.0 | Gal4 (O2) |
| Sia6 (O9) | 3.3 | Glc1 (O6) |

**GT1b internal interactions bridged by water**

| **GT1b sugar atom** | **Distance (Å)** | **Bridging Water** | **Distance (Å)** | **GT1b sugar atom** |
| --- | --- | --- | --- | --- |
| Gal2 (O2) | 2.8 | 3397 | 2.8 | Sia6 (O7) |
| GalNAc3 (N2) | 2.8 | 3394 | 2.9 | Gal4 (O2) |
| Sia5 (O8) | 3.1 | 3373 | 3.2 | Sia6 (O4) |
